# Short-Read NGS of a Long-Range *CYP21A2* Amplicon as an Improved Alternative to Sanger Sequencing for Congenital Adrenal Hyperplasia Genetic Testing

**DOI:** 10.3390/ijms27156938

**Published:** 2026-08-02

**Authors:** Zoia Antysheva, Viktor Bogdanov, Ekaterina Rutkovskaya, Anna Stepanova, Anton Esibov, Julia Krupinova, Victoria Shchekina, Ekaterina Avsievich, Tatyana Frolova, Erzhena Bazarova, Elena Demina, Evgenia Sharibzhanova, Natalia Bodunova, Elena Petryaykina, Ekaterina Petriaikina, Aleksey Ivashechkin, Yulia Katcaran, Anastasia Bukhanova, Vladimir Yudin, Anton Keskinov, Sergey Yudin, Dmitry Svetlichnyy, Mary Woroncow, Veronika Skvortsova, Pavel Volchkov

**Affiliations:** 1 Moscow Center for Advanced Studies, Kulakova Street, 20, Moscow 123592, Russia; 2 Federal Research Center for Innovator and Emerging Biomedical and Pharmaceutical Technologies, Baltiyskaya Street, Moscow 125315, Russia; 3 Moscow Clinical Scientific Center N.A. A.S. Loginov, Novogireevskaya Street, 1, Moscow 111123, Russia; 4 Russian Children’s Clinical Hospital, Leninsky Prospekt, 117, Bldg. 1, Moscow 119571, Russia; 5 Federal State Budgetary Institution «Centre for Strategic Planning and Management of Biomedical Health Risks» of the Federal Medical and Biological Agency (Centre for Strategic Planning, of the Federal Medical and Biological Agency), Pogodinskaya Street, 10, Bld. 1, Moscow 119121, Russia; 6 Faculty of Medicine, Lomonosov Moscow State University, Moscow 119991, Russia; 7 The Federal Medical Biological Agency (FMBA of Russia), Volokolamskoye Shosse, 30, Moscow 123182, Russia

**Keywords:** CAH, 21-Hydroxylase deficiency, CYP21A2, short-read NGS sequencing, Sanger sequencing

## Abstract

The standard-of-car and genetic testing for 21-hydroxylase deficiency congenital adrenal hyperplasia (CAH) is Sanger sequencing of a long-range *CYP21A2* amplicon combined with multiplex ligation-dependent probe amplification (MLPA). Many Sanger sequencing protocols capture only selected regions of *CYP21A2* and require several sequencing reactions. This retrospective study explores the potential of short-read next-generation sequencing (SRS) to replace Sanger sequencing in CAH genetic testing. A total of 216 participants, including CAH patients, their parents and individuals with no family history of CAH, were included. We assessed the performance of standard SRS analysis and improved upon it with developed computational software AmpliconPipe, which includes copy number calling and caller harmonization for precise variant detection. With these procedures, SRS of the *CYP21A2* long-range amplicon achieved 100% sensitivity (CI 95% [94.9, 100]) and 100% specificity (CI 95% [97.4, 100]) for CAH diagnosis, and 100% sensitivity (CI 95% [94.9, 100]) with 98.6% specificity (CI 95% [95.0, 99.6]) for CAH-carrier diagnosis, with better gene coverage outperforming the standard-of-care method, discovering novel variants, and detecting *CYP21A2* copy number alterations with high sensitivity. These findings establish SRS as a practical, accurate, and immediately implementable alternative to Sanger sequencing for CAH genetic testing.

## 1. Introduction

Congenital adrenal hyperplasia (CAH) is a group of inherited disorders, causing various degrees of failure in cortisol and aldosterone production up to life-threatening conditions. The incidence of severe CAH forms (salt-wasting and virilizing) varies across populations from 1:10,000 to 1:20,000, while less severe non-classical CAH incidence is estimated to be from 1:200 to 1:1000 [1,2]. Classical CAH can be detected early by elevated 17-OHP levels in blood; this test is included in neonatal screening in a number of countries, usually possessing high sensitivity and low positive predictive value (~10%) due to the 17-OHP threshold set to maximize the former [1,2,3]. A second test is often made to confirm the diagnosis and increase positive predictive value, and confirmation with different 17-OHP measurement strategies is discussed as well [4,5]. Genetic confirmation of CAH or exclusion of other rare steroidogenic diseases is often required as well as genetic consulting for further family planning [1,6].

Up to 95% of CAH cases are caused by variants in the *CYP21A2* gene [7,8]. This gene has a highly homologous pseudogene, *CYP21A1P*, and is situated in the highly repetitive and complex RCCX locus [9]. Hotspot pathogenic variants in *CYP21A2* arise from microconversions and chimerizations with *CYP21A1P* [9,10], and the copy number of the gene ranges from one to occasionally four, and the pseudogene copy number from zero to four–five [11,12]. This greatly impedes genetic testing for 21-Hydroxylase deficiency CAH (21-OHD) confirmation and further family counseling. 

The current standard-of-care method for 21-OHD diagnosis is PCR-based Sanger sequencing of a long-range *CYP21A2* amplicon coupled with multiplex ligation-dependent probe amplification (MLPA) on *CYP21A2*- and *CYP21A1P*-specific probes [6]. If pathogenic heterozygous variants are present, their cis/trans configuration is elucidated by parent analysis. However, due to the length limit of Sanger sequencing (~1000 bp), several reactions are necessary to cover the entire *CYP21A2* [13,14,15,16]. Common primer combinations target regions around the hotspot sites and do not cover the entire *CYP21A2* gene or its promoter, which results in underdiagnosis of 21-OHD CAH [6,16]. The number of sequencing reactions required and the limited scalability of Sanger sequencing also result in an increase in analysis times and costs. Additionally, multiple deletions and insertions can lead to incorrect chromatogram interpretation, and MLPA may be confounded by conversions from gene to pseudogene [16]. 

An increasing number of researchers propose usage of targeted long-read sequencing (LRS) for CAH diagnostics and specifically *CYP21A2* and *CYP21A1P* sequencing to discern all types of locus variability [16,17,18,19,20,21]. The method possesses the benefit of high-quality diagnostics along with confident read-based phasing of variants, allowing up to 100% concordance with classical Sanger + MLPA method-detected pathogenic variants in resolved cases and up to 100% diagnosis in cases of CAH that were unresolved with the classical method [16,20]. LRS can also explain heritability in families with chimeric duplications [19]. 

However, currently few clinical laboratories possess clinically validated long-read sequencing capabilities and the required personnel training [22]. Requirements for DNA amount and quality may differ from SRS, processing is less automated and requires more manual labor, and not all data analysis tools are of clinical grade yet [17,22,23]. *CYP21A2*-targeted LRS data processing is also not straightforward, requiring read filtering based on presence of certain primer pairs, which invites design and validation of a software for clinical use of this system [16,20].

Targeted short-read sequencing (SRS) presents a cheaper and immediately available alternative to *CYP21A2* LRS. Several studies have already demonstrated the viability of detecting *CYP21A2* variants from long-range *CYP21A2* amplicons with SRS [24,25,26]. However, to our knowledge, no systematic comparison of targeted SRS to the standard-of-care Sanger + MLPA method has been made, and an assessment of carrier detection or pathogenic compound resolution was not conducted either. Additionally, variant calling methods frequently are not described or not compared [21,24,25] and there is no openly available software specialized in targeted SRS quality assessment for complicated regions like *CYP21A2* or detecting duplications and deletions of a single long-range amplicon. Like LRS, SRS allows for read-based phasing; however, as expected, phased blocks are on average shorter [27] and it is unclear if standalone read-based phasing in targeted SRS can be utilized for reliable CAH diagnosis. Hence there is a need for systematic comparison of SRS capabilities, variant calling procedures, and computational software specialized in targeted SRS processing. 

This retrospective study applied SRS of *CYP21A2* long-range amplicon to the set of 216 individuals including CAH patients, their relatives, and individuals with no symptoms or family history of CAH. We developed an optimized, open-source pipeline for analyzing such data along with extra software for *CYP21A2* copy number analysis in long-range amplicons. The optimized pipeline was compared with standard methods of analyzing SRS data and with simulated Sanger + MLPA method results for CAH and CAH-carrier diagnosis accuracy. This study would be of use to clinicians and geneticists wishing to enhance CAH diagnostics within the current capabilities and setups of the clinical laboratories. 

## 2. Results

### 2.1. Study Cohort

**Figure 1:**
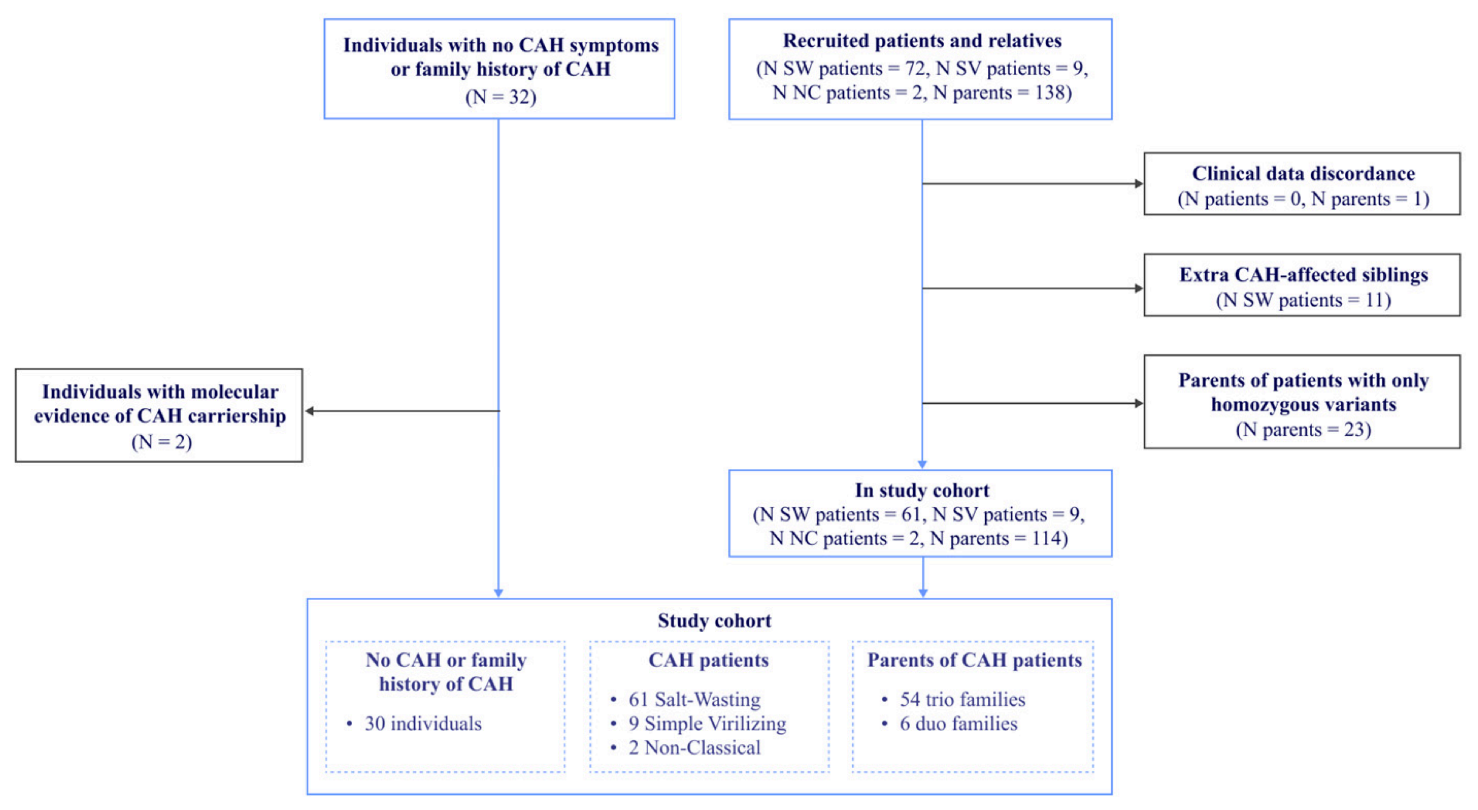
Workflow of recruitment and selection of patients, their parents, and individuals with no family history of CAH for study cohort along with CAH forms of the patients in the study cohort (SW: salt-wasting, SV: simple virilizing, NC: non-classical).

We recruited 72 families that had at least one CAH patient and at least one parent of the proband; patients with rarer simple virilizing or non-classical forms were preferred when available. Only one affected sibling per family was included. A total of 138 parents of these patients were available as well; modeling EMQN guideline recommendations, parents of patients that had only homozygous variants according to later SRS and MLPA analysis were not included in the study cohort. After filtering (Methods: Study Cohort Design), the study cohort included 72 non-related patients (sixty-one salt-wasting, nine simple virilizing, two non-classical) and 114 of their parents. Additionally, 30 individuals with no family history of CAH, CAH diagnosis or molecular evidence of CAH carriership in further analysis were included as negative control. Overall, 216 individuals were included in the study cohort (Figure 1, Table S1).

**Figure 2:**
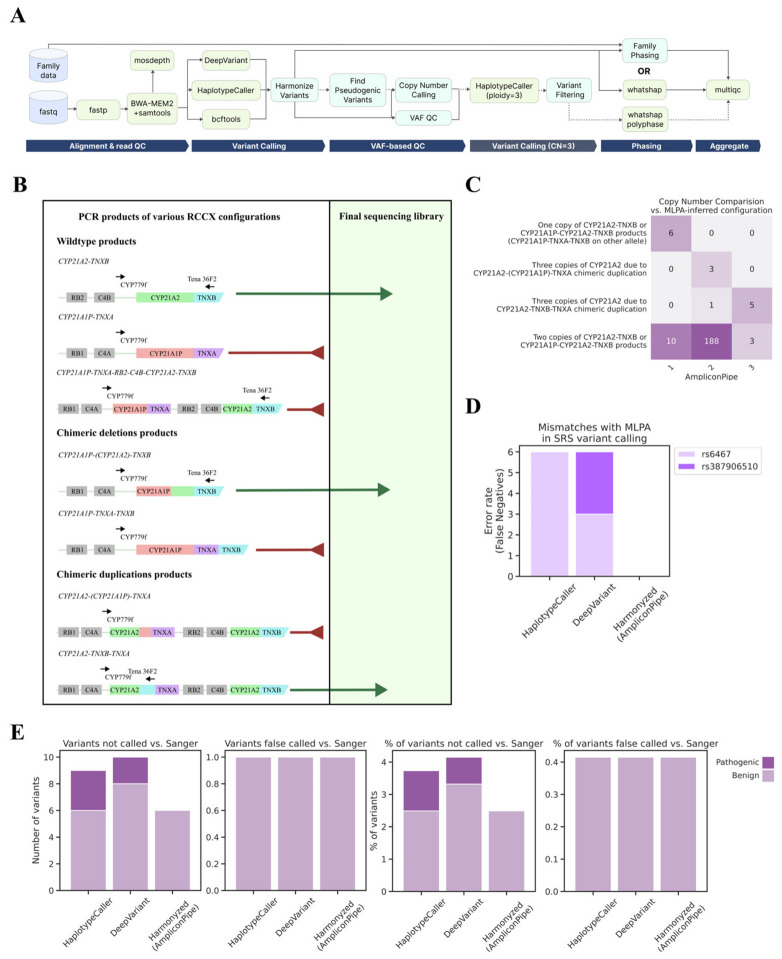
(**A**) AmpliconPipe workflow. Green: components implementing existing tools, light blue: components implementing our custom procedures, darker blue: input data. Solid line: universal procedures, dashed lines: optional procedures. (**B**) Long-range *CYP21A2* amplicon primers on possible RCCX locus configurations (not to scale). From top to bottom: wild-type *CYP21A2-TNXB* (normal gene configuration), wild-type *CYP21A1P-TNXA* (normal pseudogene configuration), wild-type *CYP21A1P-TNXA-RB2-C4B-CYP21A2-TNXB* (full locus normal configuration), chimeric deletion products *CYP21A1P-(CYP21A2)-TNXB* and *CYP21A1P-TNXA-TNXB* (results of 30 kb deletion in between pseudogene and gene, not affecting or affecting *TNXB* depending on breakpoint), and chimeric duplications *CYP21A2-(CYP21A1P)-TNXA* and *CYP21A2-TNXB-TNXA* (results of conversion of gene sequence to pseudogene, not affecting or affecting *TNXA* depending on breakpoint). Arrows indicate wherever there will be a PCR product that continues to the next library preparation stage (green is presence of a PCR product, red is absence). (**C**) Comparison of RCCX locus configurations inferred from MLPA results with AmpliconPipe copy number calling. (**D**) Comparison of MLPA pathogenic variant calls with SRS variant calling. X axis: number of errors (false negatives only), Y axis: caller, colors: variant ID. (**E**) Comparison of Sanger sequencing vs. SRS variant calling on 30 samples. Number of false-negative variants (**left**), number of false-positive variants (**left middle**), percentage of false-negative variants to all variants detected in Sanger (**right middle**), percentage of false-positive variants to all variants detected in Sanger (**right**) colored by pathogenicity of variants.

All samples in the study cohort underwent short-read sequencing of *CYP21A2* long-range amplicon (Figure 2A,B) and MLPA of CYP21A2 gene, and a set of 30 samples, including patients, parents, and negative controls underwent Sanger sequencing as well. 

### 2.2. Validation of Developed Algorithms for Targeted SRS Analysis

To facilitate precise analysis of *CYP21A2*-targeted sequencing and extract additional information, we developed a pipeline for analysis of such data, AmpliconPipe. Briefly, AmpliconPipe performs trimming and alignment, coverage quality control, then calls variants via three different callers and harmonizes resulting calls into a joint set of high-quality variants. These variants undergo relatedness-based (if family sequencing data are given) and read-based phasing to assess their cis/trans configuration. Variant allele frequency (VAF)-based quality control and copy number calling are conducted; if duplication (e.g., a copy number of three is detected) alternative calling with ploidy 3 is implemented, variants are filtered, and also phased (Figure 2A; Methods: Targeted sequencing analysis). 

VAF-QC procedures identified 11 samples (5.1%) with possible pseudogene contamination or unequal allele amplification (Figure S1). After manual inspection, nine of these eleven samples were considered problematic; their libraries were re-prepared, resequenced, and the new data replaced the originals in the dataset. Resequenced samples successfully passed quality checks, demonstrating that VAF deviations were indeed caused by technical factors. On average, 4.33 new variants were detected by HaplotypeCaller in resequenced samples compared to problematic samples, while problematic samples did not have any unique variants.

We evaluated the ability of VAF-based copy number calling to detect chimeric deletions and duplications (Figure 2B). Chimeric deletions occur due to 30 kilobase deletion between *CYP21A1P* and *CYP21A2* (or long conversion from gene to pseudogene) and result in *CYP21A1P-CYP21A2-TNXB* or *CYP21A1P-TNXA-TNXB* products. The latter effectively causes complete deletion of the gene and is not captured with *CYP21A2* long-range amplicon due to absence of Tena 36F2 (or equivalent *TNXB*) primer site. However, their presence can be inferred from VAF-based copy number analysis. Chimeric duplications are caused by conversions from gene to pseudogene and result in *CYP21A1P* sequence partially or fully replaced with *CYP21A2* sequence. Possible chimeric duplication products are *CYP21A2-CYP21A1P-TNXA* and *CYP21A2-TNXB-TNXA*. The latter is not captured with *CYP21A2* long-range amplicon primer, but does not contain full wild-type *CYP21A2* copy and is not relevant for diagnosis, while the former is captured and also can potentially be discerned with VAF-based copy number analysis. This assessment is crucial for correct genotyping of CAH patients and for detecting carriers among relatives.

*CYP21A2* MLPA results, commonly used to detect *CYP21A1P-TNXA-TNXB* products (Methods: *CYP21A2* MLPA), were compared with AmpliconPipe copy number calling. AmpliconPipe identified 6 out of 6 such deletions in the cohort; however, specificity was 95.2% (CI 95% [91.5, 97.4]) and precision was six correct calls out of sixteen (Figure 2C). Sensitivity and precision, however, were not powered enough for reliable estimates. A total of 100% precision in VAF-based deletion calling is unattainable for short regions, since it is possible that all variants match in both alleles, resulting in a fully homozygous variant set. VAFs of such a set will be identical to VAFs of a fully hemizygous one. Due to this we adopted MLPA for confirming any deletions found in targeted SRS and utilize this mode, termed optional MLPA, for further analysis. 

For *CYP21A2-TNXB-TNXA* chimeric duplications, five out of six duplications were detected and the specificity was 98.6% (CI 95% [95.9, 99.5]) (Figure 2C). MLPA analysis also singled out three samples with *CYP21A2-(CYP21A1P)-TNXA* chimeric duplications present. As expected, all these samples had a copy number of 2 on SRS, with clear VAF peaks at 0.5, due to the *TNXB* primer in the long-range amplicon specific for *TNXB* but not for *TNXA* sequence (Figure 2B,C). 

We also tested the SRS capability to detect 30 kb deletions between *CYP21A1P* and *CYP21A2*, which result in chimeric *CYP21A1P-CYP21A2* products (Figure 2B). Commonly, in long-range *CYP21A2* amplicon, 30 kb deletion is inferred from the presence of three pseudogenic variants, c.-113G>A (rs1246774295), c.-110T>C (rs909177624), c.-103A>G (rs573835051), in the gene promoter [28,29]. Since SRS captures a whole long-range amplicon, we expanded this list of variants to all variants in the promoter region (chr6:32037977-32038320) that are pseudogenic-derived (Table S2). A total of 117 out of 125 chimeras had all of these variants present; the remaining eight had all except one present. In all eight cases, the missing variant was c.-195G>C (rs3130676), indicating the possibility for populational variation in the promoter of *CYP21A1P*. We compared SRS data to 30 kb deletion MLPA data. All samples that had 30 kb deletion were confirmed by MLPA, matching in the presence and number of chimeric copies, except for one sample, which had two chimeric *CYP21A1P-CYP21A2* products according to MLPA and three according to SRS. 

The variant harmonization algorithm was validated on *CYP21A2* pathogenic variants covered by MLPA probes (Table S3) in samples with a copy number of two or less (both by MLPA and AmpliconPipe). The only errors observed among the tested callers (HaplotypeCaller and DeepVariant) were false negatives. Six variants were discordant between MLPA and HaplotypeCaller alone, six variants were discordant between DeepVariant and MLPA alone, and no discordances were observed between harmonized variants and MLPA results (Figure 2C and Figure S2A). 

c.293−13C>G (rs6467) SNP, which is situated near the site of c.332_339del (rs387906510), was the most frequently discordant. HaplotypeCaller failed to call homozygous rs6467 in the presence of homozygous rs387906510 in six out of fifteen cases where this combination occurred. DeepVariant failed to call heterozygous rs6467 in the presence of heterozygous rs387906510 or heterozygous rs387906510 in the presence of heterozygous rs6467 six times out of fifty-six.

As additional validation of SRS and variant harmonization, we performed Sanger sequencing [13] of six *CYP21A2* regions (Table S4) in 30 samples, including patients, parents, and participants with no history of CAH (Table S1). This allowed us to assess variant harmonization performance on a broader set of variants (Figure 2E). Variant harmonization achieved a minimal false-negative variant rate of 2.49% compared to standalone callers and reached a total accuracy of 99.3%. HaplotypeCaller demonstrated three and DeepVariant four unique false-negative calls, which included pathogenic rs6467 (Table S5). Six false-negative calls in harmonized variants were common for all callers (Table S5, Figure S3), demonstrating that the harmonization strategy correctly resolved all conflicts between callers and only variants absent in both were false negatives when compared to Sanger. The false-positive rate was negligible, with only one false call across 30 samples, demonstrating high fidelity of SRS. 

### 2.3. SRS Discovers Novel Variants in CYP21A2

**Figure 3:**
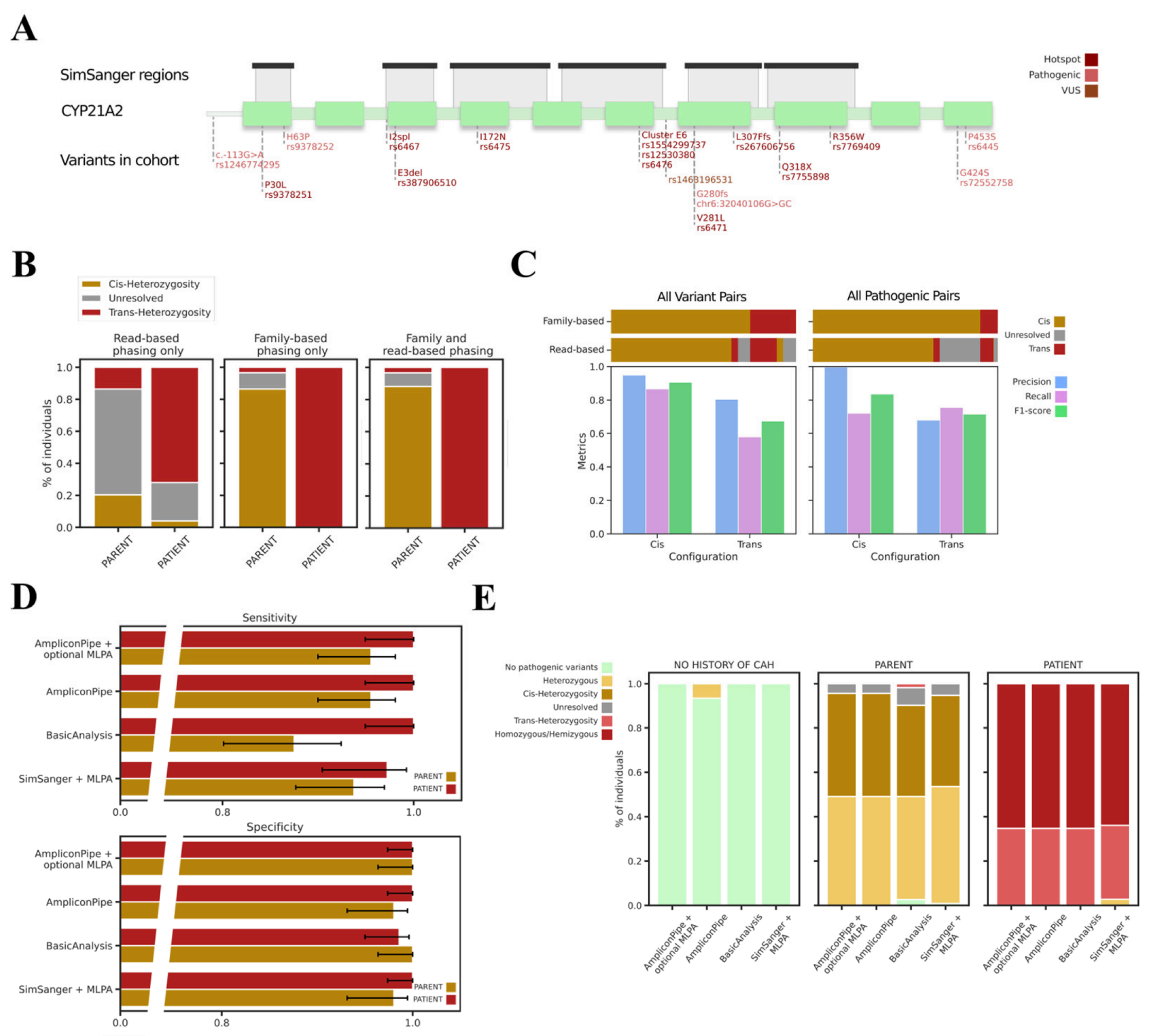
(**A**) Variants identified in the study cohort. Top: regions captured by common Sanger sequencing primers (black: region including primer sequence, gray: only captured sample sequence). Center: *CYP21A2* gene (not to scale), broad patches: gene exons, narrow: introns, the narrowest: promoter. Bottom: variants in cohort. Color identifies variants as hotspot, pathogenic, or VUS. (**B**) Status assignment based on phasing results in the phasing comparison cohort, read-based phasing only (**left**), family-based phasing only (**center**), combination of family-based and read-based (**right**). (**C**) Quality assessment of variant pairs configuration prediction by read-based phasing only. Confusion matrix for all variant pairs (**top right**), precision, recall, f1-score for all variant pairs (bottom right), confusion matrix for all pathogenic pairs (**top left**), precision, recall, f1-score for pathogenic variant pairs (**bottom left**). (**D**) Specificities and sensitivities of methods in identifying patients (should be CAH-affected) and parents (should be CAH-carriers). (**E**) *CYP21A2* genotypes detected in the study cohort groups by four methods.

We compiled a full list of pathogenic and VUS *CYP21A2* variants detected by SRS in the study cohort and intersected it with the regions that would be captured by common Sanger sequencing primers [13]. All eleven hotspot pathogenic variants arising from chimerization or conversions from the pseudogene were detected in the study cohort (Table 1). These variants would be detected with a common Sanger sequencing setup as well (Figure 3A) and the promoter pathogenic variant rs1246774295 would also be detected in this setup. 

Outside pseudogene conversions, we detected rarer pathogenic variants rs9378252 (associated with NC), rs6445 (associated with NC), and rs72552758 (associated with SW) [7,30,31,32,33,34,35]. We also discovered the pathogenic novel variant c.841dup (chr6:32040106G>GC), a frameshift in exon 7 of *CYP21A2*, which in conjunction with deletion of another *CYP21A2* copy, occurred in a patient with the SW form. Finally, a strong VUS variant rs1463196531 was detected in a patient with the salt-wasting form. This variant, which we described earlier [36], was present as heterozygous in one of the parents as well (the other parent had deletion of *CYP21A2* copy inherited by the patient), was the only potential causative variant, and was in silico predicted as causing alternative splicing. 

Variants rs6445, rs72552758, and rs1463196531 fall outside the recommended Sanger regions, which can result in underdiagnosis of CAH. rs6445 is associated with NC-CAH, making its detection essential for CAH confirmation in patients with ambiguous symptoms. Alternative primers that capture all of the *CYP21A2* exons and exon–intron bounds would be able to resolve the issue of rs6445 and rs72552758; however, these still would miss rs1463196531 in intron 6, making sequencing of the entire *CYP21A2* gene and its promoter region desirable. 

### 2.4. Optimal Phasing Strategies for Determining Variant Configuration in CYP21A2 Amplicon SRS

According to EMQN guidelines [6], sequencing of parents is required to genotype patients with two or more heterozygous variants. We assessed whether read-based phasing alone could resolve patient or carrier status in SRS data. To this end, we selected 25 patients with two or more heterozygous variants (and no homozygous or hemizygous variants) and 59 parents with two or more heterozygous variants—i.e., participants whose genetic status would be ambiguous without phasing. This comparison cohort included members of fifty-seven families, fifty-one of them trio and six duo. Read-based phasing was conducted for each participant separately; for phasing based on family data, all available parents were used. Samples with a copy number of three, families with patients with a copy number of three, or all parents with a copy number of three were not phased in family-based phasing. 

With read-based phasing, most participants remained unresolved (53.6% of the comparison cohort: six patients and thirty-nine parents) (Figure 3B and Figure S4A). Considering unresolved samples as negatively labeled in both CAH-affected and carrier prediction, read-based phasing achieved a sensitivity of 72.0% (CI 95% [52.4, 85.7]) and 86.4% (CI 95% [75.5, 93.0]) specificity for CAH-affected patients. Among participants predicted as trans-heterozygous, eighteen were true patients and eight were parents (Figure S4A). Carrier prediction quality was low: sensitivity 20.3% (CI 95% [12.0, 32.3]), specificity 96.0% (CI 95% [80.5, 99.2]), and most of the parents (66.1%) remained unresolved.

Family-based phasing demonstrated much better results, assigning status to 100% of patients and 89.8% of parents in the comparison cohort (Figure 3B and Figure S4A). Sensitivity and specificity for CAH-affected were 100.0% (CI 95% [86.7, 100.0]) and 96.6% (CI 95% [88.5, 99.0]) and for carriers they were 86.4% (CI 95% [75.5, 93.0]) and 100% (CI 95% [86.7, 100.0]). Two parents’ predicted statuses were discordant with the expected (cis-configuration and thus non-affected carrier). One parent had rs6467+rs6445/rs7755898 trans-configuration (same in read-based phasing) and another had rs72552758/rs7755898 trans-configurated (unresolved in read-based phasing). Their corresponding probands did not have rs7755898 in both cases. Allele-specific sequencing was conducted for these parents; despite not achieving full specificity because of the small number of discerning variants for primer design, allele-specific VAF-enrichments confirmed the trans-configuration of variants (Figure S4B), excluding recombination or false-negative call of rs7755898 in probands. Instead, this demonstrated the capability of SRS and family-based phasing to single out participants with previously undiagnosed CAH. 

Out of six unresolved participants, four were unresolved due to possession of three copies. The remaining two were parents of the same CAH patient. One of the parents had heterozygous rs6467, rs6471; the other had heterozygous rs9378251, rs6467, rs387906510, and rs6471. The patient inherited all of these variants, but only heterozygous rs6471, implying cis-configuration of rs6471 and previously undiagnosed NC-CAH in one of the parents. However, which parent was affected was ambiguous with family-based phasing. The parent with rs9378251+rs6467+rs387906510 had rs387906510 cis-configurated with rs6471 according to allele-specific sequencing (Figure S4B), suggesting that the other parent had rs6467/rs6471 configuration. However, since that parent did not have enough variants different between alleles for allele-specific sequencing, we did not re-classify this parent as CAH-affected.

We also assessed the performance of combining family-based and read-based phasing (the latter used when family phasing was not applicable). In this case, only five participants remained unresolved, thus classifying one sample more than only family-based phasing. This strategy yielded the best, although only marginally better, specificities and sensitivities for CAH-affected and carrier diagnosis (Figure 3B and Figure S4A, Table S6), making it the default strategy for AmpliconPipe phasing. 

To further assess the accuracy of read-based phasing, we selected 159 individuals (patients and parents) whose haplotypes had been determined by family-based phasing and who carried at least two heterozygous variants (5611 phased heterozygous variants, 91.0% of all heterozygous variants in these samples). In these samples the number of variants in phased blocks in read-based phasing was 5952, but 42.2% of samples had more than one phased block, indicating that haplotypes were not fully resolved (Figure S4C). 

We compared read-based and family-based phasing results for 140,768 heterozygous variant pairs whose configuration was determined by family-based phasing. Read-based phasing resolved only 86.5% of heterozygous variant pairs that were resolved with family-based phasing (Figure 3C and Figure S4D). F1 of cis-configuration prediction was 0.91 and f1 for trans-configuration was 0.67. Among pathogenic and strong VUS variant pairs, 85.0% were resolved, and f1 for cis- and trans-configurations were 0.84 and 0.72, respectively, further illustrating that SRS read-based phasing in amplicon data is not sufficient for correct diagnosis. 

### 2.5. Heredity and Exact Chimeric Junctions Resolved with SRS

AmpliconPipe successfully resolved pathogenic haplotypes (full configuration of pathogenic variants in sample) for 94.6% (53 out of 56) of patients with at least one heterozygous variant; no de novo pathogenic variants in patients were detected in the study cohort. Out of 127 resolved alleles in patients, including those with all homozygous variants, 63 had more than one pathogenic variant (Table 2). Most compounds, as expected, consisted of pseudogenic-derived variants due to chimeras and conversions; no compound with only non-pseudogenic variants was detected.

Based on resolved haplotypes, we also identified the presence of 68 chimeras, coupled with 30 kb deletion, in 50 CAH patients. While our study cohort did not encompass all possible chimeric types, we identified CH-1, CH-3, CH-5, CH-6, and attenuated deletion chimera types [28,29], the most frequent of them being CH-1 type (Table 3). Additionally, *CYP21A1P-TNXA-TNXB* chimeras could be suggested in three patients, where one copy was predicted and confirmed; however, since the location of breakpoint was beyond long-range PCR regions, the exact junction could not be resolved. 

SRS pinpointed chimeric junctions to the interval between the last pseudogenic variant and the first non-pseudogenic variant in a list of differences between the gene and pseudogene. CH-1 chimeras and CH-6, commonly identified by junction site rs387906510 ^ rs6475 and junction site rs6467 ^ rs387906510, were split into two subcategories. Attenuated chimeras in our cohort did not belong to a known type (CH-4, CH-9), but instead presented a novel junction site at rs9378251 ^ rs6463 (Table 3).

### 2.6. SRS Is More Sensitive to Both CAH-Affected and Carriers, While Maintaining High Specificity

For overall comparison of diagnostics capabilities, we selected three SRS scenarios and the standard-of-care Sanger + MLPA one. The first SRS scenario was AmpliconPipe, the second was AmpliconPipe coupled with MLPA for deletion conformation (AmpliconPipe + optional MLPA). The third was a representation of SRS standard analysis (BasicAnalysis), namely, read trimming, alignment, coverage assessment, variant calling with one caller (HaplotypeCaller), and logical phasing with family data (Figure S5A). All tools used in BasicAnalysis were identical to ones used in AmpliconPipe. Since VAF-based quality control is an AmpliconPipe feature, original sample data before resequencing were used. 

The standard-of-care method was represented with a simulation of results that would be derived from standard-of-care Sanger + MLPA combination (SimSanger + MLPA). MLPA was done for all samples, while the Sanger set of variants was simulated from a harmonized high-quality variant set (all samples assumed to have a copy number of two) from AmpliconPipe by intersecting said variant set with regions captured by common *CYP21A2* Sanger sequencing primers (Figure 3A) [13]. As we demonstrated above on a subset of 30 samples, SRS was highly concordant with Sanger sequencing and no pathogenic variants were discordant, allowing the use of harmonized variant set to proxy Sanger sequencing results in all study cohort samples. This reflects the genomic coverage advantage of SRS versus Sanger sequenced regions, but does not reflect Sanger-specific mistakes and biases, which can also negatively impact variant calling. Family data was used for pathogenic variant phasing in samples that did not have *CYP21A2-TNXB-TNXA* chimeric duplication according to MLPA (Figure S5B).

Diagnostic capabilities were compared by rate of correct diagnosis of CAH patients and carriership in parents of said patients, as well as absence of CAH in individuals with no family history of CAH. Two parents for whom trans-configuration of pathogenic variants and thus previously undiagnosed CAH were suspected above were excluded from comparisons (metrics for cohort without exclusion of these participants are given in Figure S6 and Table S8). 

All SRS-based methods demonstrated 100.0% (CI 95% [94.9, 100]) sensitivity in CAH patient diagnosis, while SimSanger + MLPA sensitivity to CAH patients was lower, 97.2% (CI 95%: [90.4, 99.2]) (Table S9). SimSanger + MLPA labeled two CAH patients as carriers due to pathogenic variants outside of common Sanger regions (Figure 3A,C and Figure S7) that were the only causative variants on their alleles. In our cohort, rs6445 occurred only in compounds with other variants (Table 2) and its absence in SimSanger regions did not cause any misclassifications; however, cases when it does occur separately would also be misclassified. 

Both AmpliconPipe scenarios achieved the highest CAH-carrier sensitivities (95.5%, CI 95% [90.0, 98.1]), followed by SimSanger + MLPA (93.8%, CI 95% [87.7, 97.0]), missing a carrier of a variant outside of SimSanger regions. BasicAnalysis demonstrated the lowest CAH-carrier sensitivity, 87.5% (CI 95% [80.1, 92.4]). Since BasicAnalysis could not detect copy number changes, it classified all parents who carried only one copy of *CYP21A2* as non-carriers, resulting in three mislabeled individuals (Figure 3C and Figure S6). Additionally, in three families, HaplotypeCaller’s failure to call homozygous rs6467 in CAH-affected probands led to rs6467 variants remaining unphased (five parents) or incorrectly assessed as trans-configurated (one parent), resulting in unresolved and false-positive CAH-affected statuses, respectively. In one CAH-affected proband, BasicAnalysis did not detect heterozygous rs9378251 in a sample flagged as problematic and resequenced in AmpliconPipe scenarios (in the resequenced sample, the variant was detected by HaplotypeCaller), which led to misclassification of the parent as trans-configurated in family-based phasing. 

Both the AmpliconPipe scenarios and the SimSanger + MLPA method achieved the highest specificity for CAH patient diagnosis: 100.0% (CI 95% [97.4, 100]). For BasicAnalysis, the CAH patient specificity was lower (98.6% (CI 95% [95.0, 99.6]) due to two carriers being misclassified as trans-configurated as described above.

Carrier specificity was the highest for AmpliconPipe + optional MLPA and BasicAnalysis 100.0% (CI 95% [96.4, 100.0]), while AmpliconPipe scored 98.0% (CI 95% [93.1, 99.5]). This occurred due to false-positive deletion detection in non-carriers in AmpliconPipe, while MLPA deletion confirmation rectified the issue. BasicAnalysis had no deletion detection and yielded no false positives, although at the cost of decreased sensitivity as described above. SimSanger + MLPA carrier specificity was 98.0% (CI 95% [90.3, 99.0]) due to two CAH patients misclassified as carriers. 

Comparison of scenarios’ predictions with McNemar’s test revealed statistically significant (*p*-value < 0.05) differences between both AmpliconPipe scenarios and BasicAnalysis in predicting CAH-carriers (Table S10). Other comparisons were not significant due to small numbers of divergent participants; however, all participants divergent between methods had clear molecular causes for the divergence, indicating that the difference will reach significance in larger cohorts. Sequencing of regions beyond common hotspots is vital for detection of rarer *CYP21A2* pathogenic variants, and SRS sequencing of long-range amplicons can provide a reliable and inexpensive way to do so.

## 3. Discussion

The current standard-of-care method for diagnosing 21-OHD CAH is Sanger sequencing combined with MLPA [6]. However, covered regions are of limited length and several sequencing reactions are required, which increases costs and processing times, while leaving some CAH cases underdiagnosed [6,13,16]. In this retrospective study, we explored the capability of short-read sequencing of long-range *CYP21A2* amplicons to replace the current method. We evaluated SRS performance in CAH patients, carriers, and unaffected individuals, measuring specificity, sensitivity, and the ability to resolve heredity in families. To enhance SRS analysis, we developed an open-source software, AmpliconPipe, for quick amplicon processing and extra quality control. While this study focused on its applications to *CYP21A2* and CAH diagnosis, AmpliconPipe can be applied to any amplicon and its capabilities for VAF-based quality control may be used for other genes that possess highly similar pseudogenes, like SMN1/SMN2. 

AmpliconPipe achieved 100% sensitivity (CI 95% [94.9, 100]) and 100% specificity (CI 95% [97.4, 100]) for CAH diagnosis, successfully identifying both classical and non-classical CAH. Notably, all SRS scenarios (including BasicAnalysis) also demonstrated 100% sensitivity and the ability to detect new and rare variants outside hotspot regions. These include rs1463196531 (previously classified as a VUS but shown to be the only causative variant on a patient’s second allele [36]) and 32040106G>GC, a novel frameshift insertion in exon 7. Our study was also enriched in carriers to assess the performance of SRS (for AmpliconPipe carrier SE: 95.5%, CI 95% [90.0, 98.1], SP: 100.0% (CI 95% [96.4, 100.0]) in genetic counseling scenarios, where discerning carriers from CAH-affected is of importance. SRS of long-range amplicon demonstrated high concordance with Sanger sequencing, with AmpliconPipe demonstrating the best accuracy (99.3%) in all variants and 100% concordance in pathogenic variants. SRS also provided detailed chimeric breakpoint resolution, which can be of use in population studies of *CYP21A2*, providing more insight into RCCX recombinations.

An important benefit of the proposed method, compared to LRS, is that it requires minimal changes to existing *CYP21A2* genetic analysis workflows. Long-range PCR amplification of *CYP21A2* is already performed prior to Sanger sequencing [13], and MLPA is already part of the current standard of care. Since short-read next-generation sequencing is already widely used in clinical laboratories [37,38], transitioning to SRS of long-range PCR products would require minimal changes. The cost of library preparation (e.g., using the Nextera XT kit, Illumina) and sequencing of a single long-range *CYP21A2* amplicon at 500× coverage (2 × 300 bp) can be estimated at $42 [39,40]. By comparison, a single Sanger sequencing reaction covering 200–300 bp along with preceding PCR typically costs $5–10 [41,42]. Because comprehensive *CYP21A2* analysis requires multiple sequencing reactions (12 in the protocol described by Lee et al. [13]), the total cost of Sanger sequencing may reach at least $60 per sample. 

Sanger sequencing also requires substantially longer processing time than NGS. Although high-throughput 96-capillary instruments are available, most academic and clinical laboratories operate 16- or 24-capillary sequencers, which require approximately 4–6 h to process a 96-well plate—eight *CYP21A2* amplicons. Depending on the sequencing chemistry and the sequencer, a full NGS run requires approximately 18–56 h [40]. However, a single flow cell can accommodate up to 500–1500 amplicons depending on the kit. Consequently, despite the longer runtime of an individual sequencing run, NGS becomes more time-efficient than Sanger sequencing when processing over 50 *CYP21A2* amplicons simultaneously.

After NGS sequencing, although standard SRS analysis can be used, in our study such an approach (BasicAnalysis) performed worse than AmpliconPipe because of caller-specific errors and the lack of copy number calling. While the former did not impact CAH-affected diagnostics due to mistakes occurring only in conjunction with other pathogenic variants, it did affect parents’ genetic assessment. We found that the usage of more than one caller and consequent harmonization of calls are optimal for capturing all of pathogenic variants in SRS. Copy number calling, as described here, can be used to determine RCCX locus configuration and to subsequently call variants with the correct ploidy. Alternatively, MLPA can be used as in the standard-of-care Sanger + MLPA setup.

In our study cohort, AmpliconPipe SRS analysis demonstrated performance on par with targeted LRS retrospective studies (targeting other CAH-associated genes as well [17] or *CYP21A2-*only [20]). Our study had a comparable or larger number of probands [16] than these studies, but also included CAH-carriers and negative controls for better method capability assessment. 

The limitations of targeted short-read sequencing are as follows: first, the need to sequence at least one parent for reliable phasing. We demonstrated that standalone read-based phasing of SRS is insufficient for correct diagnosis and the usage of parent data is still recommended.

Second, VAF-based copy number detection (any implementation, not AmpliconPipe only) cannot achieve 100% specificity for deletions (*CYP21A1P-TNXA-TNXB* products) on short regions. This can cause false-positive carrier calls, but no false-positive affected calls, since the presence of any homozygous pathogenic variant marks an individual as affected, while the presence of a heterozygous pathogenic variant excludes *CYP21A2* deletion. False-positive carrier calls arise when an individual possesses two identical *CYP21A2* haplotypes without pathogenic variants. In our study, two individuals out of the participants without family history of CAH were false-positive carriers; however, the number of such participants was not sufficient for reliable frequency estimates. Additionally, the frequency of false-positive events is dependent on population haplotype frequencies and will vary in different cohorts. The rarity of chimeric duplications (*CYP21A2-TNXB-TNXA*) in our cohort also resulted in unreliable confidence intervals for sensitivity and specificity estimates. Still, SRS potentially reduces the number of samples that require MLPA to 11.1% and, consequently, brings down analysis cost per sample. 

Another limitation of copy number detection, this time specific to AmpliconPipe procedures, is that the maximal copy number tested is three. Potential cases with a copy number of four will be flagged as contaminated; this configuration, however, has been described in singular cases and is very rare. MLPA can be used if such a configuration is suspected. 

The absence of the duplication product *CYP21A2-CYP21A1P-TNXA* (which arises from gene-to-pseudogene conversion and is not captured by the long-range CYP21A2 amplicon primers) might appear to be a limitation. However, this product does not possess a full wild-type sequence of *CYP21A2* and its promoter and thus will be irrelevant for CAH diagnosis. Even if a wild-type *CYP21A2* copy is present (e.g., in a *CYP21A2-TNXA* chimeric duplication), it is also likely to be non-functional because the *CYP21A1P* upstream region contains *C4A*, but not *C4B*. *C4B* intron contains *CYP21A2* distal regulatory loci (ZB) essential for gene expression [43,44,45], while the ZA sequence in *C4A* may act as a CYP21 expression suppressor [46]. Mutations in *C4B* have been reported to attenuate *CYP21A2* expression [47,48]. There was a described CAH case with 30 kb deletion, not affecting the *CYP21A2* promoter, but removing *C4B* [49]. Consequently, product *CYP21A2-(CYP21A1P)-TNXA* might not provide an expression of a functional copy. Meanwhile, extra copies of *CYP21A2-TNXB* may arise from gene to pseudogene conversion or from true duplication of the whole RCCX module [11,50,51]. The former may be irrelevant for diagnosis similarly to *CYP21A2-TNXA*; the latter, however, can provide a fully functional copy. The long-range *CYP21A2* amplicon is limited with not being able to differentiate these rare configurations; however, this is not specific to SRS but is a general long-range *CYP21A2* amplicon limitation, the same for the standard-of-care method and targeted LRS. Another limitation would be rare configuration combinations, where one allele possesses a full chimeric deletion of the *CYP21A2* copy, while the other allele possesses a *CYP21A2* copy and chimeric duplication as well. Such a configuration will present itself as a normal copy number of two on SRS. However, the same again would be true for the standard-of-care and targeted LRS method, not being specific to SRS.

While PCR may introduce mistakes into sequencing data, which has been listed as a limitation of long-range PCR applied to *CYP21A2* [52], in our study no false-positive CAH-affected or carriers occurred due to this factor. Additional filtering offered in AmpliconPipe ensured the removal of dubious variants and complete concordance with the MLPA results. Uneven allele amplification is also a possible downside of long-range PCR [53,54], but VAF quality analysis coupled with MLPA for suspected deletions allows for the detection of such samples.

Overall, this retrospective study shows that targeted short-read sequencing of *CYP21A2* could serve as a reliable and immediate replacement for Sanger sequencing as a standard-of-care method, which, coupled with MLPA for deletion confirmation, was able to achieve total 97.7% accuracy in individual classification with MLPA and 96.8% without MLPA. Our cohort possessed a number of probands comparable to or exceeding retrospective targeted LRS studies [17,18,20] and was additionally enriched in carriers, allowing us to demonstrate affected vs. carrier status assignment quality. Additional modules, such as VAF-based copy number calling and quality control, increase the amount of information that can be extracted from targeted SRS. SRS combined with AmpliconPipe enhances both the specificity and sensitivity of CAH and CAH-carrier detection, allowing for rare variant discovery, precise family counseling, and population studies. Further directions include applications to other highly homologous genes, and larger validation studies for capability assessment in neonatal screening.

## 4. Materials and Methods

### 4.1. Ethical Approval

This study was approved by the local ethical committee of OOO NCMI “Universimed” (Protocol №14, date of approval 21 July 2023) and was conducted in accordance with the principles of the Declaration of Helsinki. Written informed consent was obtained from all participants or their legal representatives prior to enrollment in the study.

### 4.2. Enrollment of Participants and Sample Collection 

The study included patients with a genetically or biochemically confirmed CAH diagnosis of any form, as well as parents of those patients. Patients were eligible if they had available biochemical workup data at CAH manifestation (sodium and potassium), and 17-Hydroxyprogesterone (17-OHP) levels at manifestation and/or neonatal screening. CAH diagnosis was assigned in concordance with Russian Federation clinical recommendations [55,56]. Briefly, for children, the main criterion was doubly confirmed 17-OHP elevation during neonatal screening combined with the presence of CAH clinical symptoms. In adults, non-classical CAH was also diagnosed with doubly confirmed 17-OHP basal level elevation (>30 nmol/L) and, optionally, the presence of symptoms, including acne, hirsutism, menstrual cycle disorders, or miscarriages. Participants aged 0–99 years were eligible. Patients with the rarer simple virilizing or non-classical forms were preferentially recruited when available, to avoid a bias towards the salt-wasting form (which could be overrepresented because patients with severe CAH have more frequent contact with clinical endocrinology centers). Exclusion criteria were confirmed human immunodeficiency virus (HIV), hepatitis B or C, syphilis infections, other forms of primary adrenal insufficiency, a participant’s refusal to continue participation in study, and (or) informed consent form withdrawal. 

Patients and relatives were recruited through clinical endocrinology centers in Moscow and by targeted informing in relevant internet communities between August 2023 and September 2025. 

From all participants, 6 mL of peripheral venous blood was collected by venipuncture in EDTA-containing tubes followed by DNA extraction. All samples were screened for hemolysis and lipemia, and were transported under constant temperature control. 

### 4.3. Study Cohort Design

A total of 83 patients with clinically diagnosed CAH from 72 families were recruited, with preferential recruitment of patients with the rarer simple virilizing or non-classical forms when available. A total of 138 parents of these patients were recruited as well. Patients were checked for clinical data concordances (biochemical and clinical data corresponding to the classical form in patients diagnosed with non-classical CAH and vice versa would be considered discordant and such patients excluded). Parents’ records were checked for absence of CAH diagnosis. One parent was found to have contradictory records about absence/presence of CAH diagnosis and was excluded. In 9 families, there was more than one CAH patient among siblings. Extra patients were randomly excluded so that each family in the study cohort would have only one proband. Modeling EMQN guideline recommendations, parents of patients who had only homozygous variants (according to subsequent SRS and MLPA analysis) were not included in the study cohort. Overall, 72 non-related patients (61 salt-wasting, 9 simple virilizing, 2 non-classical) and 114 of their parents were selected (Figure 1). 

Control samples were obtained from established collections at the “Centre for Strategic Planning and Management of Biomedical Health Risks”. A total of 32 participants without symptoms or family history of CAH were extracted from different collections. After further analysis of SRS amplicon data, 2 of them demonstrated evidence of CAH carriership and were excluded to ensure a homogenous reference set in the study cohort. A total of 30 participants were included in the study set.

### 4.4. CYP21A2 MLPA 

*CYP21A2* MLPA analysis was conducted using the MLPA Probemix P050-D1 CAH kit (MRC-Holland, Amsterdam, the Netherlands). as per manufacturer instruction. Interpretation was conducted as per manufacturer instruction. Additionally, the CYP21A2-wt-113 probe was used to assess presence of 30 kb deletion leading to *CYP21A1P-CYP21A2* chimerization. A number lower than 2 copies of the probe signified presence of 30 kb deletion.

*CYP21A2* overall copy numbers were determined as follows. A sample was labeled as possessing a full deletion of a *CYP21A2* copy, if only one non-pseudogenic copy was detected in all *CYP21A2* probes and at least one following *TNXB* probe was affected. 

If at least two consecutive *CYP21A2* probes had a copy number of 3 (other downstream probes could have a copy number of 2) and no *TNXB* probe had a copy number of 3, we inferred the presence of the chimeric product *CYP21A2-TNXA*, which arises from gene-to-pseudogene conversion. If some probes in the start of the *CYP21A2* had 2 copies, while further probes had 3 copies, chimeric duplication coupled with conversion from pseudogene to gene and/or chimeric deletion on another allele was inferred.

When more than at least two consecutive probes of *CYP21A2* had a copy number of 3 and at least two consecutive probes of *TNXB* had a copy number of 3, presence of chimeric product *CYP21A2-TNXB-TNXA* was inferred, also stemming from conversion from gene to pseudogene. If all *TNXB* probes were amplified, presence of the whole duplicated RCCX module, instead of conversion from gene to pseudogene, would be inferred. If some *TNXB* probes had 3 copies, while some or all of the *CYP21A2* probes had less than 3 copies, chimeric duplication coupled with conversion from pseudogene to gene and/or chimeric deletion on another allele was inferred.

### 4.5. CYP21A2 Long-Range Amplicons Preparation and Sequencing 

The genetic analysis of *CYP21A2* was performed by amplicon sequencing. Genomic DNA was isolated from whole-blood samples using the Tecan Freedom EVO automated station (Tecan, Zurich, Switzerland) using the MagAttract HMW DNA Kit (Qiagen, Hilden, Germany). The concentration and purity of the isolated DNA was determined on an Infinite F Nano+ tablet reader (Tecan, Zurich, Switzerland), and the quality assessment was performed using NanoDrop 8000 (Thermo Fisher Scientific, Walham, MA USA). 

Long-range PCR products were generated with *CYP21A2*-gene-specific primers, according to the protocol previously described [13]. The amplicons were subjected to sequencing using the Illumina MiSeq platform (Illumina, San Diego, CA, USA) with the v3 reagents kit, generating paired-end reads of 2 × 300 bp. The average amplicon sequencing depth was more than 2000×.

### 4.6. Targeted Sequencing Analysis with AmpliconPipe 

Raw reads were trimmed with fastp v0.23.1 and mapped to the *CYP21A2* amplicon sequence (chr6:32037620-32043828, UCSC GRCh38/hg38, coordinates of long-range CYP779f-Tena 36F2 amplicon [13] were extracted with UCSC In-Silico PCR tool) by Burrows-Wheeler Aligner software (BWA-MEM2, v2.2.1) [57,58], then deduplicated with samtools v1.23 [59]. Mate-fixing was conducted with samtools. Mosdepth v0.3.3 was used to assess coverage. Minimal coverage was set as 100×; samples with coverage below the threshold were redone. 

HaplotypeCaller v4.5.0.0 with default settings [60], DeepVariant v1.5.0 in WES mode as the closest available mode to short-read amplicon data [61], and bcftools v1.23 with options -L 20000, --no-BAQ, -d 3000 [59] were used for variant calling. Variants from different callers were harmonized as follows: (1) if a variant was called identically by both HaplotypeCaller and DeepVariant, it was kept unchanged. (2) If the variant was present in only one of those callers or the genotype was discordant, the following checks were applied. For a HaplotypeCaller variant (if present), the depth should be higher than 30, VAF in bounds from 0.25 to 0.75 for the heterozygous genotype and from 0.75 to 1 for the homozygous. If the variant was exclusive for DeepVariant or the HaplotypeCaller check was not passed, bcftools calling results were used. Resulting variants were merged in the final vcf. 

Variants were phased with whatshap v2.8 [62]. Alternatively, if relatedness data were available, family-based phasing was performed by identifying all possible combinations of the patient’s and parents’ genotype configurations for each variant separately. If only one possible inheritance combination existed for a variant, phasing configuration was recorded; with two combinations, the variant was left unphased; with zero combinations, the variant was left unphased and marked as a potential de novo variant. If only one parent was available or one parent had a copy number of 3, the proxy parent (assumed to be heterozygous for all variants) was used. If inheritance of a variant was possible from both the real and proxy parent, the real parent was preferred.

Copy number calling (in the range from 1 to 3) and assessment of pseudogene admixture was conducted (Methods: VAF-based copy number prediction and quality control). If the predicted copy number was equal to 3, alternative variant calling was conducted with HaplotypeCaller with the ploidy set as 3. Resulting variants were filtered by depth (>30) and by VAF with following bounds: from 0.1 to 0.495 for genotype 0/0/1, from 0.495 to 0.85 for genotype 0/1/1, and from 0.85 to 1 for genotype 1/1/1. Remaining variants were phased with the whatshap polyphase option. 

The final quality report was aggregated with multiqc v1.31 [63].

The pipeline was implemented with snakemake v7.32.4. VAF-based copy number detection, quality control, and variant harmonization were implemented in Python v3.12. Containerization was done in docker v29.1.3 or singularity v3.8.7; the pipeline supports both versions of containers. 

### 4.7. VAF-Based Copy Number Prediction and Quality Control

To create a universal list of variants that differ between the pseudogene and the gene and would be detectable in SRS alignment data after BWA mapping, we simulated reads from the pseudogenic sequence (*CYP21A1P*, chr6:32005689-32008399, UCSC GRCh38/hg38) using NEAT v3.4. [64]. NEAT was run with paired-end mode with fragment mean length of 150, deviation 30, coverage 100, zero random mutation, and zero error rates. Generated reads were aligned to the *CYP21A2* amplicon reference sequence with BWA-MEM2. Variants were called with HaplotypeCaller. 

For the copy number prediction and quality control, the sample’s VAF of variants was extracted from filtered VCF. Variants were split into pseudogenic and non-pseudogenic sets according to the list formed above. The minimum number of variants in the set was 3; otherwise variants were not split. If the total number of variants was less than 3, copy number estimate and quality control were not conducted.

Gaussian Mixture Model (scikit-learn, v1.7.0 [65]) was fitted on the VAF of variants for each set. The best number of components in the range from 1 to 4 (4 being the next number after 3 VAF peaks expected for a copy number of 3) was chosen according to the Bayesian information criterion. Resulting components whose means differed by ≤0.2 (so that peaks of copy number 4 will not be merged) and whose 10-standard-deviation intervals overlapped by ≥30% were merged. After merges, components that had less than 3 variants were removed as noise. If no components remained in either variant sets, variant sets were merged and fitted and filtration procedures were repeated. If a combined set also failed, copy number estimate and quality control were not conducted further. Otherwise, components were tested for correspondence to VAF peaks expected from different copy numbers (peaks with means of 1/*n*, 2/*n* … *n*/*n*, where *n* is the tested number of copies). A peak was accepted as matching to a component if the component’s mean was within 10 standard deviations of the component and within 0.085 VAF of the expected mean; 0.085 is the half of distance between heterozygous peaks for copy number 2 and copy number 3, ensuring the match is not called if the peak is closer to the location of the peak in the other copy number. If all components had a matching unique peak, the corresponding copy number was accepted. For a copy number of one, we also checked for the absence of heterozygous variants (VAF between 0.25 and 0.75). If no copy number from 1 to 3 fit, contamination was considered. 

Results of the fits on variant sets were combined. The sample was considered possibly contaminated if any of the sets returned a possible contamination. If both sets of variants returned the same copy number, it was accepted. Additionally, if a copy number equal to one was accepted for any set of variants, an additional fit, filtration, and copy number testing procedure was conducted to determine the final copy number. If copy number predictions differed between the set of variants, the highest was accepted.

### 4.8. Variant Annotation in CYP21A2

Pathogenic variants in *CYP21A2* were curated using the following framework. Variants were identified through structured interrogation of the ClinVar database, complemented by a literature review via PubMed without date restrictions. Publications on *CYP21A2*-associated congenital adrenal hyperplasia were used as primary sources for variant identification and were retrieved using the PubMed Advanced Search Builder. Boolean search strategies were applied to improve specificity and sensitivity by combining gene-related terms (*CYP21A2*, CYP21, 21-hydroxylase, RCCX locus/module, *CYP21A1P*), variant-related terms (variant, mutation*, allele*, polymorphism*, chimera*, deletion*, conversion*, hybrid*), and disease-related terms (congenital adrenal hyperplasia, CAH, 21-hydroxylase deficiency, 21-OHD, 21OHD). ClinVar records were filtered to include only variants classified as Pathogenic or Likely Pathogenic according to the guidelines of the American College of Medical Genetics and Genomics (ACMG). To ensure reliability, only variants with multiple independent submitters and no conflicting interpretations were retained. For each selected variant, available supporting evidence was also reviewed and the following eligibility criteria were applied. Variants were retained if identified in individuals with biochemically confirmed 21-hydroxylase deficiency, supported by functional evidence demonstrating reduced enzymatic activity, observed in trans with a known pathogenic allele, or representing validated structural rearrangements or gene conversion events. Population allele frequencies derived from gnomAD v4.1.0 were evaluated against disease prevalence thresholds to exclude variants incompatible with recessive inheritance. Synonymous variants without functional impact and variants lacking sufficient molecular resolution were excluded. All retained variants were considered as pathogenic in further analysis.

Additionally, VEP [66] was used for annotation of variants that were not in the curated list of pathogenic variants. Pathogenic, likely pathogenic, and strong variants of uncertain significance (VUS) according to ACMG guidelines were also considered CAH-causative variants during individual status assignment.

### 4.9. Sanger Sequencing and Analysis

Genomic DNA was isolated from whole-blood samples using the Auto-Pure 96, (Allsheng, Hangzhou, China) purification system with the MagPure Universal DNA Precast Kit (Magen, Guangzhou, China). The concentration and purity of the isolated DNA was determined using Equalbit 1 × dsDNA HS Assay Kit (Vazyme, Nanjing, China) on fluorometer Fluo-800 (Allsheng, Hangzhou, China), and the quality assessment was performed using Nano-500 (Allsheng, Hangzhou, China). 

Amplification of 6 *CYP21A2* fragments was performed with 2× Vazyme LAmp Master Mix (Vazyme, Nanjing, China) as described in protocol [13]. Amplification products were analyzed on 2.5% agarose gel, subjected to enzymatic purification using ExS-Pure™ (NimaGen, Nijmegen, the Netherlands). Sequencing reaction was performed using BrilliantDye™ Terminator v3.1 Cycle Sequencing Kit (NimaGen, Nijmegen, the Netherlands), purified with iX-Pure™ DyeTerminator Clean-up Kit (NimaGen, Nijmegen, the Netherlands). Sequencing was performed on a Locus Seqtor 1616 genetic analyzer (Helicon, Moscow, Russia).

Traces were aligned with tracy align -t 5 to the reference sequences corresponding to each Sanger amplicon. Reference sequences were obtained by extracting the corresponding regions on chromosome 6 using the UCSC In-Silico PCR tool (GRCh38/hg38, primer pairs listed in Table S4). Reverse primer In3-1 (C6/In3-1 amplicon) did not exactly match chromosome 6 *CYP21A2*, instead differing by two nucleotides from the corresponding sequence in the C6 primer vicinity. The corrected primer was used for determining Sanger amplicon coordinates on hg38. Samtools was used to extract reference sequences in separate fasta files that were used for alignment. Regions of trace alignment on the reference sequence according to tracy align were extracted for each amplicon in samples; for each amplicon region covered by both forward and reverse traces was calculated.

Variants in Sanger sequencing traces were called separately for forward and reverse reads using tracy decompose -t 5 [67]. Then variants were combined and only variants that were in the regions covered by both forward and reverse according to tracy were retained. Variants discordant between forward and reverse were discarded if quality was less than 30. Remaining discordant variants were resolved by manual inspection of traces via UGENE [68] with Sanger trace alignment to corresponding reference sequences. 

### 4.10. Allele-Specific SRS Sequencing 

Genomic DNA was isolated from whole-blood samples as described in Sanger sequencing and analysis. For all three allele-specific sequenced samples, Tena 36F2 was used as the reverse primer. Forward primers were selected to be situated to be in the location of the maximal number of variants different between alleles and to have a melt temperature in the range of 5 degrees of the Tena 36F primer melt temperature according to UCSC In-Silico PCR tool (Table S7). 

Long-range *CYP21A2* amplicon PCR was conducted as described above. Purification of the product was conducted using magnetic beads in the ratio 1:0.8. Then the product was diluted to 10 ng/mcl concentration and 1 mcl of the solution was used for the second, allele-specific, PCR. The second PCR was conducted with a 2× Vazyme LAmp Master Mix kit (Vazyme, Nanjing, China) according to manufacturer instructions. Libraries prepared with “SG GM” Raissol kit (Raissol, Moscow, Russia) according to instructions and sequenced with SurfSeq 5000 (GeneMind, Shenzhen, China), generating paired-end reads of 2 × 150 bp.

Sequenced libraries were filtered and aligned as described above in Methods: Targeted sequencing analysis. Allele depths at positions of interest, extracted with Integrative Genome Viewer, were used to calculate VAFs of variants. 

### 4.11. Simulated Sanger Set Creation

Simulated Sanger variants were created as follows: harmonized variants from three callers (a copy number of two was assumed for all samples) were intersected with regions sequenced by one of the common Sanger sequencing primer combinations, which includes all hotspot pathogenic variants [13]. All variants that were in Sanger sequencing regions (Table S4) and not in primer sequences themselves were considered as such that would be detected in Sanger sequencing analysis. 

### 4.12. Statistical Analysis

Metrics were calculated with Python v3.10.14. For sensitivity and specificity calculations, individuals with an unresolved status were considered negatively labeled for both CAH-affected and carrier predictions. Wilson confidence intervals for classification metrics were calculated with statsmodels v.0.14.5. McNemar’s test (exact = False) was calculated with statsmodels v.0.14.5. Plotting was performed with seaborn v0.13.2 and matplotlib v3.10.3.

## Figures and Tables

**Table 1:** Pathogenic and VUS CYP21A2 variants of CAH patients identified in the study cohort [7,30,31,32,33,34,35].

Variant	Protein Change	dbSNP ID	Location	Associated Form	Detected in Alleles of CAH Patients *
Hotspot pathogenic variants derived from pseudogenic sequence					
c.92C>T	P30L	rs9378251	Exon 1	NC	60/134
c.293−13C>G c.-126C>G	In2G	rs6467	Intron 2	SW, SV	96/134
c.332_339del	G110Efs	rs387906510	Exon 3	SW	58/134
c.518T>A	I172N	rs6475	Exon 4	SV	28/134
c.710T>A	I236N	rs1554299737	Exon 6	SW	9/134
c.713T>A	V237E	rs12530380	Exon 6	SW	9/134
c.719T>A	M239K	rs6476	Exon 6	SW	9/134
c.844G>T	V281L	rs6471	Exon 7	NC	32/134
c.923dup	L307fx	rs267606756	Exon 7	SW	10/134
c.955C>T	Q318X	rs7755898	Exon 8	SW	8/134
c.1069C>T	R356W	rs7769409	Exon 8	SW	6/134
Other pathogenic variants derived from pseudogenic sequence					
c.-113G>A	-	rs1246774295	Promoter	NC	62/134
Non-pseudogene pathogenic variants					
c.188A>T	H63P	rs9378252	Exon 1	NC	4/134
c.841dup	G280fs	-	Exon 7	-	1/134
c.1273G>A	G424S	rs72552758	Exon 10	SW	1/134
c.1360C>T	P453S	rs6445	Exon 10	NC	3/134
Non-pseudogene VUS variants					
c.738+75C>T	-	rs1463196531	Intron 6	-	1/134

* The allele is a CYP21A2 copy present in a patient. Patients with deletions have one allele; patients with duplications have three.

**Table 2:** Pathogenic and VUS *CYP21A2* variant compounds identified in the study cohort.

Variant List	Has Non-Pseudogenic Variants	Overall Associated Form	Detected in Alleles of CAH Patients
rs1246774295, rs9378251, rs6467, rs387906510, rs6471	No	SW	27/127
rs1246774295, rs9378251, rs6467, rs387906510	No	SW	14/127
rs1246774295, rs9378251, rs6467, rs387906510, rs6475, rs1554299737, rs12530380, rs6476, rs267606756	No	SW	6/127
rs1246774295, rs9378251	No	NC	3/127
rs6467, rs6445	Yes	SV	3/127
rs1246774295, rs9378252, rs6467, rs387906510	Yes	SW	2/127
rs1246774295, rs9378251, rs6467, rs387906510, rs6475, rs1554299737, rs12530380, rs6476, rs267606756, rs7755898	No	SW	2/127
rs1246774295, rs9378251, rs6467	No	SV	2/127
rs1246774295, rs9378251, rs6467, rs387906510, rs6475	No	SW	1/127
rs1246774295, rs9378251, rs6467, rs7755898	No	SW	1/127
rs1246774295, rs9378251, rs9378252, rs6467, rs387906510	Yes	SW	1/127
rs6467, rs6471	No	SV	1/127

**Table 3:** Detailed chimeric configurations identified in the study cohort.

General Type	Classical Junction Site	Detailed Junction Site	Of All Detected Chimeras in CAH Patients
Attenuated	rs9378251 ^ rs6467	rs9378251 ^ rs6463	3/68
CH-1	rs387906510 ^ rs6475	rs193922546 ^ rs6475	48/68
CH-1* (−P30L)	rs387906510 ^ rs6475	rs397515531 ^ rs10947229	1/68
CH-1* (−P30L)	rs387906510 ^ rs6475	rs193922546 ^ rs6475	3/68
CH-3* (−V281L)	rs7755898 ^ rs7769409	rs7755898 ^ rs7769409	2/68
CH-5	rs267606756 ^ rs7755898	rs6442 ^ rs7755898	8/68
CH-6	rs6467 ^ rs387906510	rs6467 ^ rs6474	1/68
CH-6	rs6467 ^ rs387906510	rs6474 ^ rs193922545	2/68

## Data Availability

AmpliconPipe is available at https://github.com/biomedcentre/AmpliconPipe with GNU GPL v.3.0 license. Archived version as of 31 May 2026 is deposited at Zenodo: 10.5281/zenodo.20478952. Dependencies are snakemake v7.32.4 or higher, make v4.4.1 and engine (docker v29.1.3 and/or singularity v3.8.7 or higher). The data generated in this study are available upon request due to Russian legal regulations concerning privacy of genetic data and mandating order of application for sharing these data.

## Supplementary Materials

The following supporting information can be downloaded at: https://www.mdpi.com/article/10.3390/ijms27156938/s1.

## Abbreviations

The following abbreviations are used in this manuscript:

21-OHD	21-Hydroxylase Deficiency
17-OHP	17-Hydroxyprogesterone
CAH	Congenital Adrenal Hyperplasia
LRS	Long-read sequencing
MLPA	Multiplex Ligation-dependent Probe Amplification
NC	Non-classical
NGS	Next-Generation Sequencing
PCR	Polymerase Chain Reaction
SRS	Short-read sequencing
SE	Sensitivity
SP	Specificity
SV	Simple virilizing
SW	Salt-wasting
VAF	Variant Allele Frequency
VUS	Variant of Uncertain Significance
