# Supplementary material for: Short-Read NGS of a Long-Range CYP21A2 Amplicon as an Improved Alternative to Sanger Sequencing for Congenital Adrenal Hyperplasia Genetic Testing
Source: Int J Mol Sci. 2026 Aug 2;27(15):6938. doi: 10.3390/ijms27156938 (PMC13466479; doi:10.3390/ijms27156938)
Supplement: Supplementary file 1 [file ijms-27-06938-s001.zip › Figures S1-S7.pdf]

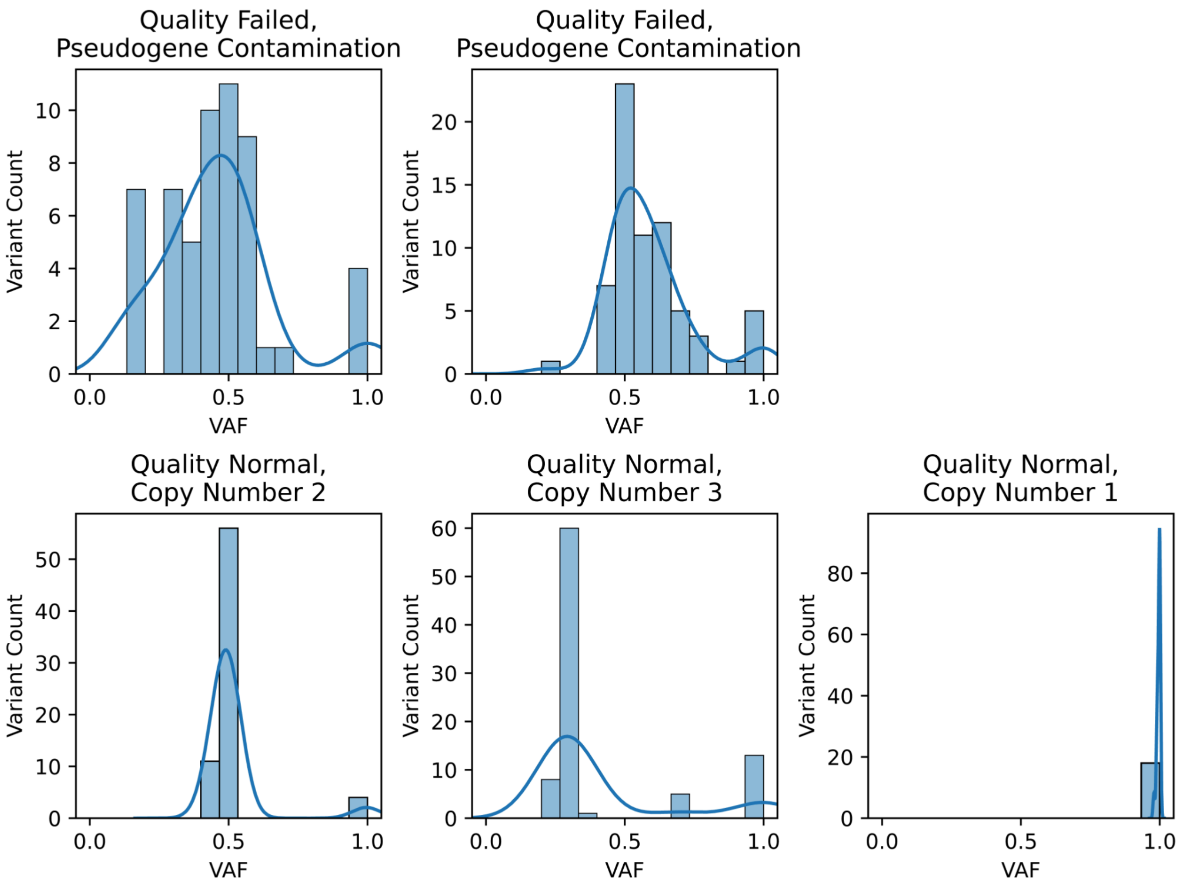

Figure S1. Examples of VAF distributions corresponding to quality failed samples (top row), and samples that passed quality checks (bottom row) and had copy number of 2 (bottom left), copy number of 3 (bottom center), and copy number of 1 predicted (bottom right).

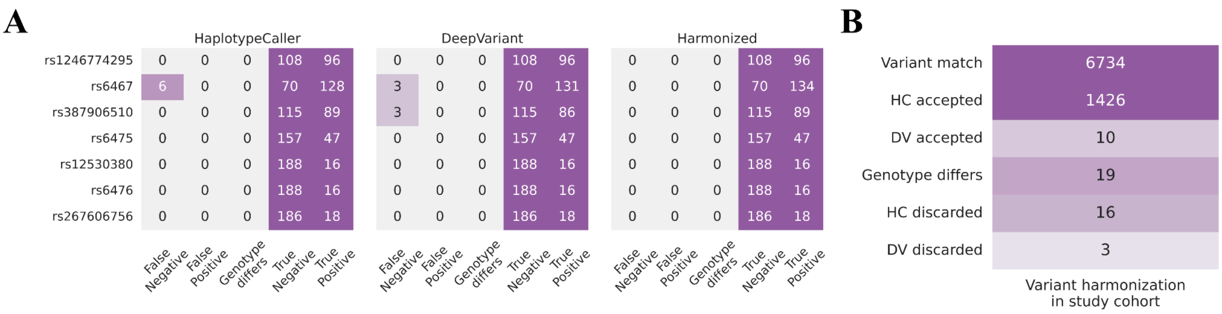

Figure S2. A. Comparison of variant callers with MLPA results on study cohort. Left - HaplotypeCaller, center - DeepVariant, right - harmonization of callers implemented in AmpliconPipe. Y axes represent dbSNP ids of SNPs corresponding to probes in MLPA, X axes represent result of caller across samples (numbers of false negatives, false positives, genotype discordances, true negatives, and true positives). **B.** Statistics of variant calling and variant harmonization compared between HaplotypeCaller and DeepVariant.

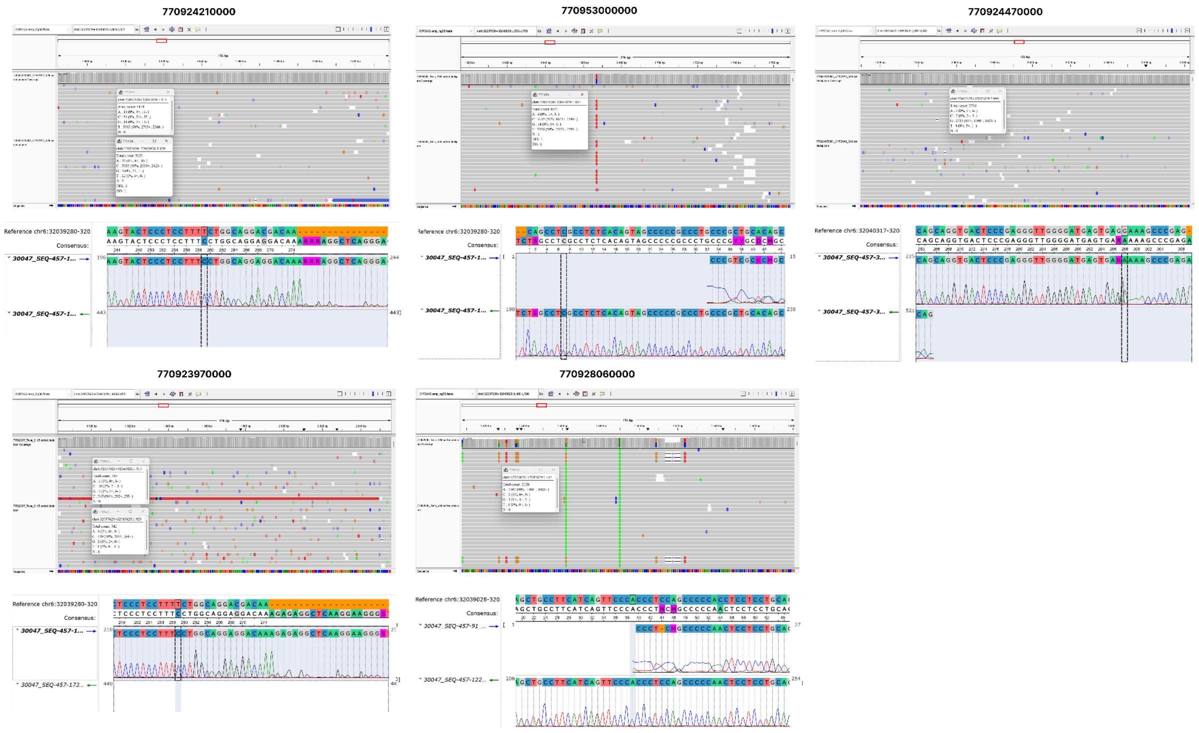

Supplementary Figure 3. IGV and UGENE (top in bottom) tracks at positions of Sanger vs. NGS discordance in samples that are discordant in all variant calling methods.

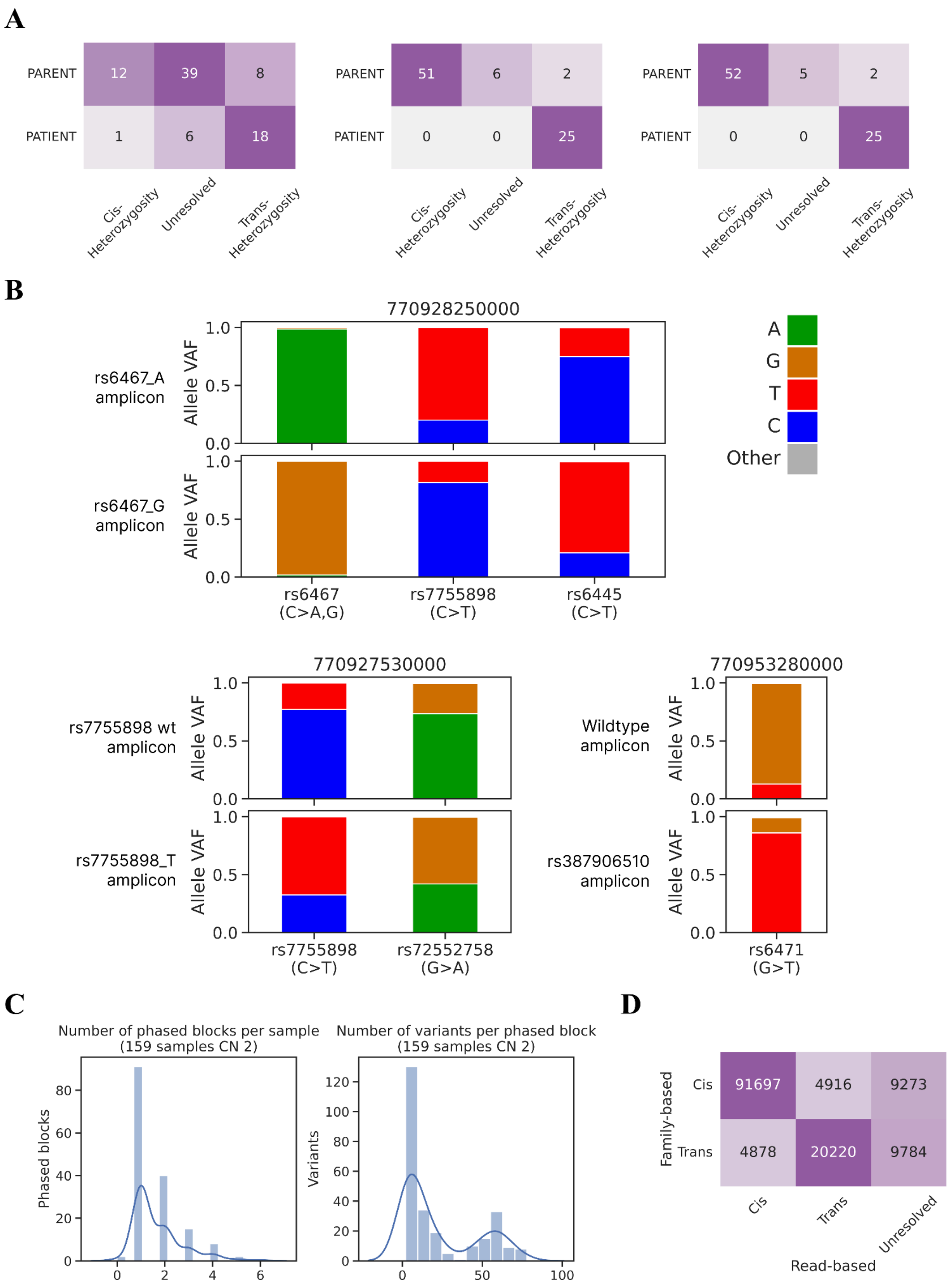

Supplementary Figure 4. A. Statistics of status assignment for phasing comparison cohort, read-based phasing only (right), family-based phasing only (center) and read-based phasing (left). **B.** VAFs of pathogenic variants of interest in allele-specific amplicons. Top: parent with trans-configuration rs6467+rs6445/rs7755898, bottom left: parent with trans-configuration

rs7755898/rs72552758, bottom right: parent with cis-configuration rs9378251+rs6467+rs387906510+rs6471. **C.** Number of phased blocks per sample distribution in samples with predicted copy number (CN) 2 (left), number of variants in phased blocks per block distribution in samples with predicted copy number (CN) 2 (right). **D.** Assignment of cis or trans configurations for all family-resolved heterozygous variant pairs with read-based sequencing.

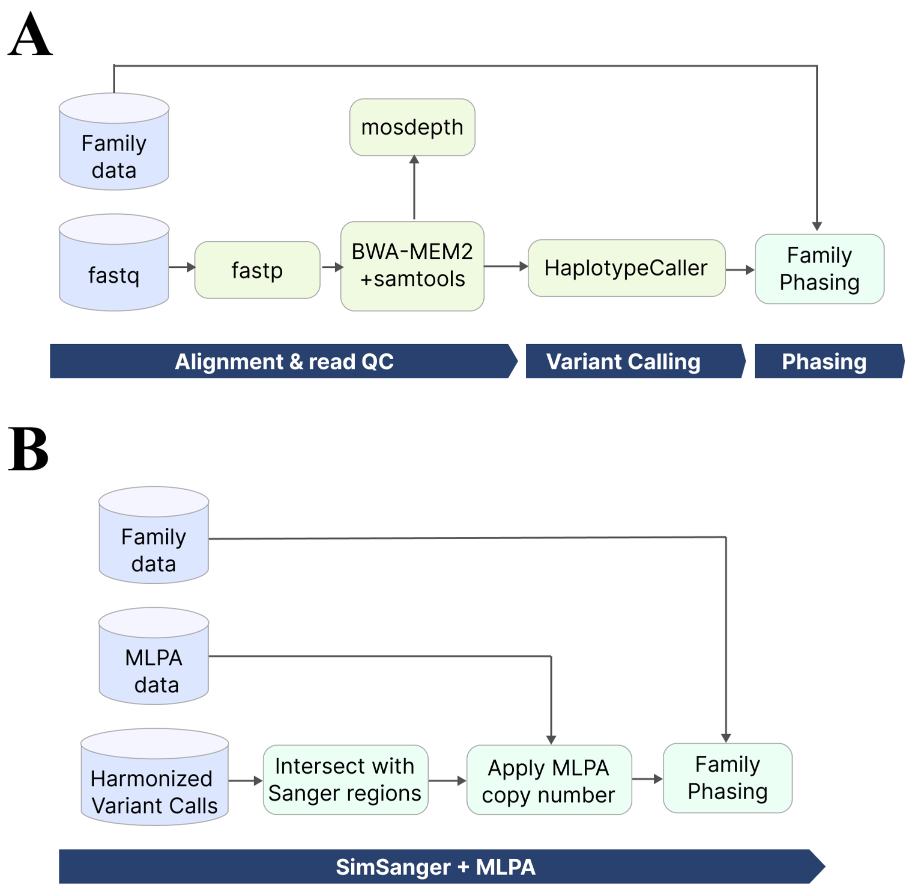

Supplementary Figure 5. A. Workflow of BasicAnalysis for targeted SRS data. Green - components implementing existing tools, light blue - components implementing our custom procedures, darker blue - input data. **B.** Workflow of simulating Sanger set of variants and combining it with MKPA. Green - components implementing existing tools, light blue - components implementing our custom procedures, darker blue - input data.

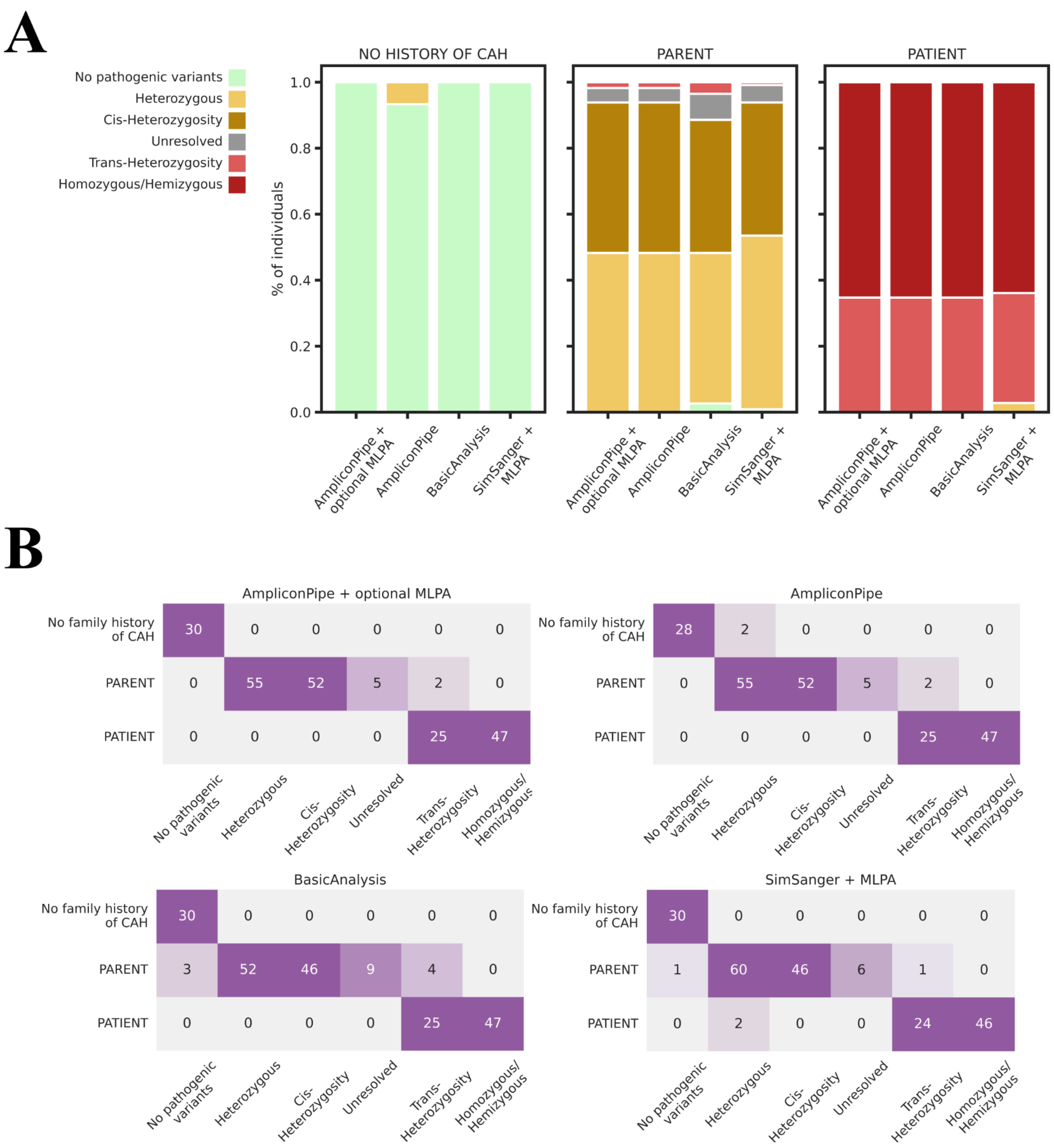

Supplementary Figure 6. A. *CYP21A2* genotypes detected in the study cohort with two trans-configurated parents by four method**s. B.** Confusion matrix of individual status in study cohort with two trans-configurated parents against *CYP21A2* genotypes detected by four methods (AmpliconPipe + optional MLPA (top left), AmpliconPipe (top right) BasicAnalysis (bottom left), SimSanger + MLPA (bottom right)).

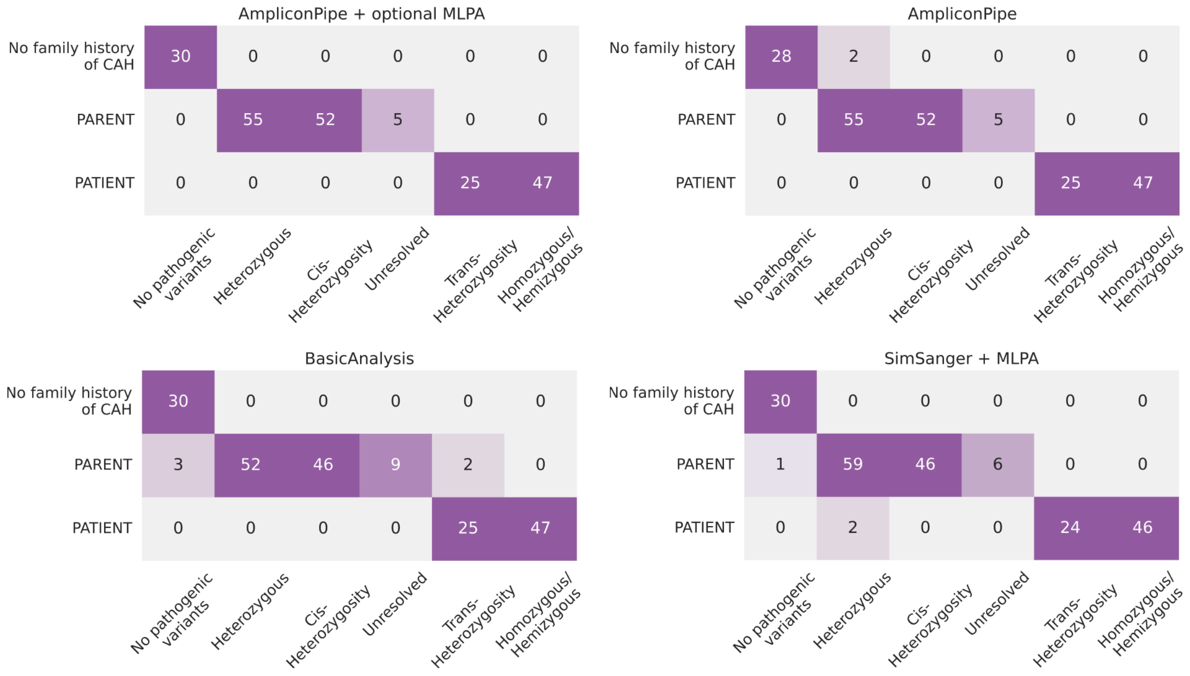

Supplementary Figure 7. Confusion matrix of individual status in study cohort against CYP21A2 genotypes detected by four methods (AmpliconPipe + optional MLPA (top left), AmpliconPipe (top right) BasicAnalysis (bottom left), SimSanger + MLPA (bottom right)).
